# Association of race and ethnicity with mortality in adults with SLE: a systematic literature review and meta-analysis

**DOI:** 10.1136/lupus-2024-001383

**Published:** 2025-02-11

**Authors:** Samir Patel, Zijing Yang, Deepak Nagra, Maryam Adas, Mark Russell, Sam Norton, Chris Wincup, James Galloway, Kate Bramham, Patrick Gordon

**Affiliations:** 1 King's College London, London, UK

**Keywords:** Mortality, Systemic Lupus Erythematosus, Epidemiology

## Abstract

**Objectives:**

Ethnicity and health outcomes are intrinsically interrelated, although mechanisms are complex. SLE is a disease with higher incidence in Asian, Black, Hispanic and Indigenous populations than in White populations. SLE is associated with premature mortality, but it is unclear if ethnicity impacts on health outcomes as studies are frequently underpowered. We aimed to describe the association between SLE and mortality across different racial and ethnic groups using meta-analysis.

**Methods:**

We identified studies of adults with SLE that reported mortality, stratified by racial and ethnic group, through a systematic literature review. We used a pairwise meta-analysis to determine the pooled odds ratio (OR) of death for those from underserved groups compared with those of White race and ethnicity.

**Results:**

Thirty-seven studies, comprising 85 578 patients with SLE, were included. Mortality was higher in Black patients (OR 1.30 (95% CI 1.16 to 1.46)) and Indigenous patients (OR 1.47 (95% CI 1.11 to 1.94)), while Asian and Hispanic patients showed no significant differences compared with White patients with SLE. Seventy per cent of included studies were conducted in the USA and when excluded, the significant difference in mortality between Black and White individuals with SLE was no longer seen (OR 0.84 (95% CI 0.54 to 1.31)).

**Conclusion:**

Overall, patients with SLE from Black or Indigenous racial and ethnic groups had higher mortality than those of White race and ethnicity. We observed no significant association in the mortality of Black patients compared with White patients from non-USA cohorts, but a scarcity of data outside of the USA was highlighted. We promote caution in the use of race and ethnicity as a factor in determining mortality risk until more generalisable data are available.

**PROSPERO registration number:**

CRD42023379034.

**WHAT IS ALREADY KNOWN ON THIS TOPIC**

- Asian, Black, Hispanic and Indigenous race and ethnicity are often identified as risk factors for mortality in SLE; however, despite multiple survival studies, no meta-analyses have been conducted to support this notion.

**WHAT THIS STUDY ADDS**

- Higher mortality was observed in Black and Indigenous racial and ethnic groups compared with White patients with SLE.
- Higher mortality was not seen for Black patients with SLE compared with White patients in studies outside of the USA.

**HOW THIS STUDY MIGHT AFFECT RESEARCH, PRACTICE OR POLICY**

- Our results revealed important geographical inconsistencies in the mortality of patients with SLE across races and ethnicities.

## Introduction

 SLE is subject to inherent health inequalities, with a higher incidence in females and in those of Black race and ethnicity compared with White race and ethnicity.^1^ In the UK, the incidence of SLE is more than four times higher in the Black Caribbean population and two times higher in the Black African population compared with the White population.^1^ Previous studies have described worse outcomes in patients who are young, newly diagnosed or of Black race and ethnicity.^2^

Historically, SLE had a strikingly high mortality rate, with an estimated 50% of patients surviving to 10 years from diagnosis in the mid-1950s. Pooled survival estimates improved to 89% at 10 years in high-income countries by 2016.^3^ Despite this positive trend, SLE continued to exhibit a 67% higher mortality rate when compared with age, gender and region-matched individuals without SLE in the UK as recently as 2012.^4^ Higher mortality in SLE is attributable to greater cardiovascular disease, renal disease, infections and certain types of cancer (eg, haematological malignancies).^2^

Clinical manifestations and outcomes are thought to vary across races and ethnicities in patients with SLE.^2^ This is particularly evident in cases of lupus nephritis, which is more frequent in Black, Asian and Hispanic patients compared with their White counterparts.^5 6^ Additionally, dogma suggests that patients with SLE of Black race and ethnicity have a higher mortality rate compared with those of White race and ethnicity. Krishnan and Hubert used national death records in the USA, from 1979 to 1998, to illustrate disproportionately higher mortality in Black versus White patients with SLE compared with those without SLE.^7^ Bernatsky *et al* further supported this by demonstrating a higher standardised mortality ratio (SMR) for Black patients compared with White patients with SLE in the USA.^2^

Race and ethnicity are dynamic and overlapping terms with varying utility and meaning across countries and generations.^8 9^ Race can be perceived as a sociopolitical construct based on physical characteristics or self-identification, while ethnicity may better represent shared cultural values, traditions and beliefs.^10^ Both race and ethnicity are widely reported in USA-based studies, whereas race has been supplanted by ethnicity coding in Europe.^8 11^ For the purpose of this review and to allow comparisons across international cohorts of patients with SLE, we refer to race and ethnicity in keeping with current recommendations.^9 12^ This raised the wider question of if we are able to appropriately risk stratify populations with blunt group labels, or whether it is the presence of nuanced non-biological factors within racial and ethnicity coding that drives associations with poor outcomes (eg, deprivation, racism, health literacy and access).^13 14^

There has not been a comprehensive review of racial and ethnic differences in the mortality of SLE to justify the preconceived notion of worse outcomes for those of Asian, Black, Hispanic or Indigenous race and ethnicity. Therefore, we aimed to investigate the association of race and ethnicity with mortality in patients with SLE. Our primary objective was to describe and quantify any differences from published studies. Secondary objectives included exploring the effect of study origin and period.

## Methods

### Search strategy and selection criteria

We performed a systematic literature search of the databases MEDLINE, Embase and the Cochrane Library, from database inception to 31 December 2023. Search terms included systemic lupus erythematosus, ethnicity, race, mortality and synonyms (full details can be found in online supplemental table S1). Our meta-analysis was prospectively registered on PROSPERO (CRD42023379034) and reported as per the Preferred Reporting Items for Systematic Review and Meta-Analysis (PRISMA) guidelines.^15^

Eligible studies were identified by two investigators (SP and ZY), who independently reviewed titles and abstracts, followed by a review of full texts of relevant articles. Study eligibility criteria were retrospective and prospective cohort studies, randomised and non-randomised controlled trials that reported on the mortality of adults >16 years old with SLE. We excluded animal studies, case reports, case series, reviews, meta-analyses, studies of discoid or cutaneous lupus erythematosus and those that did not report mortality for a White patient group. White patients with SLE were used as a uniform comparator for mortality against other racial and ethnic groups, recognising that Asian, Black, Hispanic and Indigenous race and ethnicity represented minority and underserved populations. When studies had overlapping cohorts, we included the most recently published report to avoid duplication. Disagreements were resolved by discussion to achieve a consensus.

### Data collection

Two investigators independently performed data extraction (SP and ZY). Data were collected on: year, country, data source, study type, study period, number of patients, race and ethnicity, percentage of SLE classification criteria met, average age, sex, comorbidities, medication use, SLE International Collaborating Clinics Damage Index (SDI), number lost to follow-up, number of deaths and cause of death. When outcome data were only available in graph form, we extrapolated them using a plot digitiser application (https://plotdigitizer.com/).

We recognised that race and ethnicity are considered separate entities in some countries and acknowledged both as social constructs.^9 12^ In the absence of better classifications and aligned with current reporting,^12^ we harmonised definitions of a patient’s race and ethnicity into: Asian, Black, Hispanic, Indigenous and White (box 1). Black patients included African, African American or Afro-Caribbean. In some studies, Asian race and ethnicity was subcategorised into South Asian and East Asian groups. South Asian patients were defined as originating from the Indian subcontinent. East Asian patients were those with origins from countries such as China, Japan, South Korea or Thailand. Indigenous patients were those native to the country of study (eg, Indigenous Australians or Americans). We categorised all patients who identified as Hispanic or Puerto Rican into the Hispanic group regardless of their racial identity (eg, White Hispanic or Black Hispanic), although we appreciated some studies may have coded those of Hispanic ethnicity as White or Black.

Box 1 **Definitions of race and ethnicity**

We recognised that race and ethnicity are considered separate entities in some countries and acknowledged both as social constructs. The terminology surrounding race and ethnicity has changed over generations in the literature, so in keeping with current standards, we harmonised definitions of a patient’s race and ethnicity into Asian, Black, Hispanic, Indigenous and White:

- Asian patients also included those who identified as East Asian, South Asian, Chinese, Filipino, Indian or Japanese.

- Black patients also included those who identified as African, African American or Afro-Caribbean.

- Hispanic patients were those who identified as such or Puerto Rican, regardless of their racial identity (eg, White Hispanic or Black Hispanic).

- Indigenous patients also included those who identified as native to the country of study (eg, Indigenous Australian or American).

- White patients also included those who identified as Caucasian.

Two independent reviewers (SP and DN) assessed study bias using the Newcastle Ottawa Scale for observational studies and the Cochrane Risk of Bias 2 (RoB 2) score for randomised studies.^16 17^ Observational studies were considered to have a low risk of bias if they achieved four stars for selection, two for comparability and three for ascertainment of the outcome. Studies achieving two or three stars for selection, one for comparability and two for outcome ascertainment, were considered to have a medium risk of bias. When studies achieved one star for selection or outcome ascertainment, or zero stars for any of the three categories, they were regarded as a high risk of bias.

### Statistical analysis

We calculated the unadjusted OR for mortality from extracted data. In studies with no death events, we applied a fixed continuity correction of 0.5. For the purpose of analysis, the White racial and ethnic group was considered the reference group. Pooled estimates were obtained using a random-effects model with the DerSimonian and Laird method due to the expected heterogeneity. The heterogeneity among studies was assessed by the inconsistency statistic (I^2^), where 0%, 25%, 50% and 75% indicated absent, low, moderate and high heterogeneity, respectively. Subgroup analyses were carried out by country, race and ethnicity. Meta-regression models including study duration and mid-year of each study period were fitted to explore whether these explained between study heterogeneity. We conducted two further sensitivity analyses including inception cohorts only and excluding studies with a high risk of bias. Studies of inception cohorts were considered high quality as they avoided left truncation (ie, prevalent cohorts may have missed patients that passed away prior to the study start date). Pooled estimates were presented in forest plots, ordered by the mid-year of each study period, to visually explore whether their association had changed over time. Funnel plots and Egger’s tests were used to evaluate publication bias. P value <0.05 was the level of statistical significance. All analyses were performed in R V.4.2.2 (R Project for Statistical Computing).

### Patient and public involvement

Patients and the public were not involved in the design, conduct or reporting of this research but are being involved in the dissemination of its findings.

## Results

### Study characteristics

**Figure 1:**
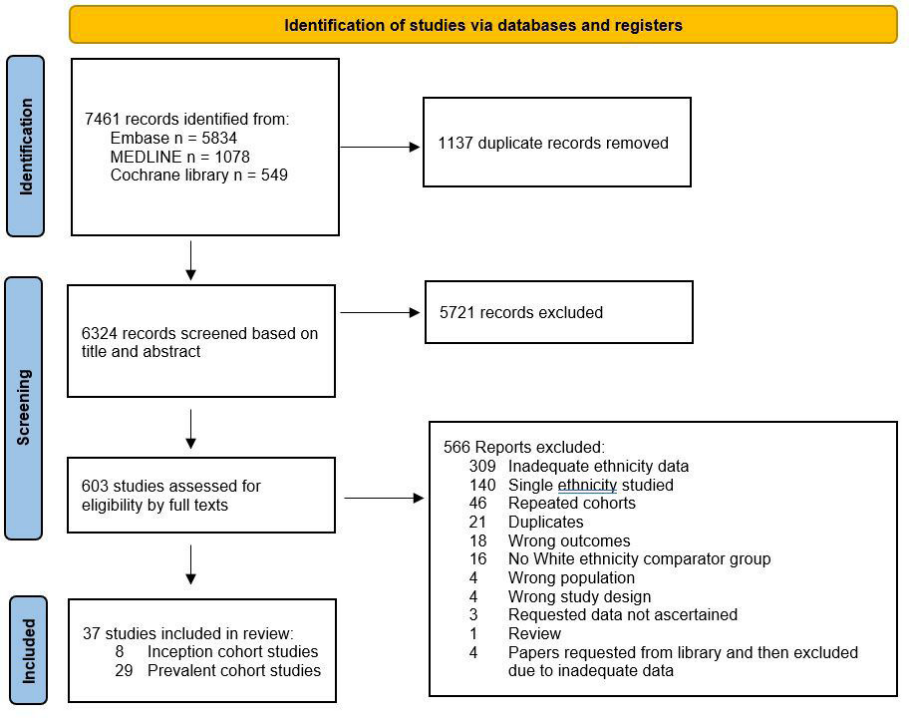
PRISMA (Preferred Reporting Items for Systematic Review and Meta-Analysis) flow diagram of literature search and study inclusion.

The systematic literature search identified 7461 titles and abstracts, 37 of which met the inclusion criteria (figure 1): 22 retrospective cohort studies, 14 prospective cohort studies and 1 randomised control trial. The earliest study started in 1949, the latest ended in 2021 and 59% had ≥10 years of follow-up. The characteristics and main findings of included studies are shown in online supplemental table S2.

A total of 85 578 patients were included, 38 971 (45.5%) patients identified as White; 32 068 (37.5%) Black; 9305 (10.9%) Hispanic; 4413 (5.2%) Asian and 821 (1.0%) Indigenous (online supplemental table S2). Thirty-two studies (86.5%) reported on Black race and ethnicity, 17 out of 37 (45.9%) Asian, 11 out of 37 (29.7%) Hispanic and 6 out of 37 (16.2%) Indigenous race and ethnicity. Most of these studies were conducted in the USA (n=26, 70%). Other countries included Australia (n=2), Brazil (n=1), Canada (n=2), South Africa (n=1) and the UK (n=3); 2 studies were multinational. Twenty-four studies (65%) satisfied time-relevant diagnostic criteria for SLE (Arthritis Rheumatism Association 1971, 1982 or American College of Rheumatology 1997), while the rest did not comment.

Where described, the average age at diagnosis was 33.7 years (SD 4.4; six studies) and 33.7 years on entry to the study (SD 6.6; nine studies). The average study duration was 13.3 years (SD 8.9, range 1–40 years).

Most individuals were female (88.6%, SD 5.9; reported in 21 studies). Only eight studies reported on comorbidities and 12 on numbers lost to follow-up. Data on cause of death, medication use, SDI and socioeconomic status were rarely reported and so could not be analysed further.

### Meta-analysis and meta-regression

**Figure 2:**
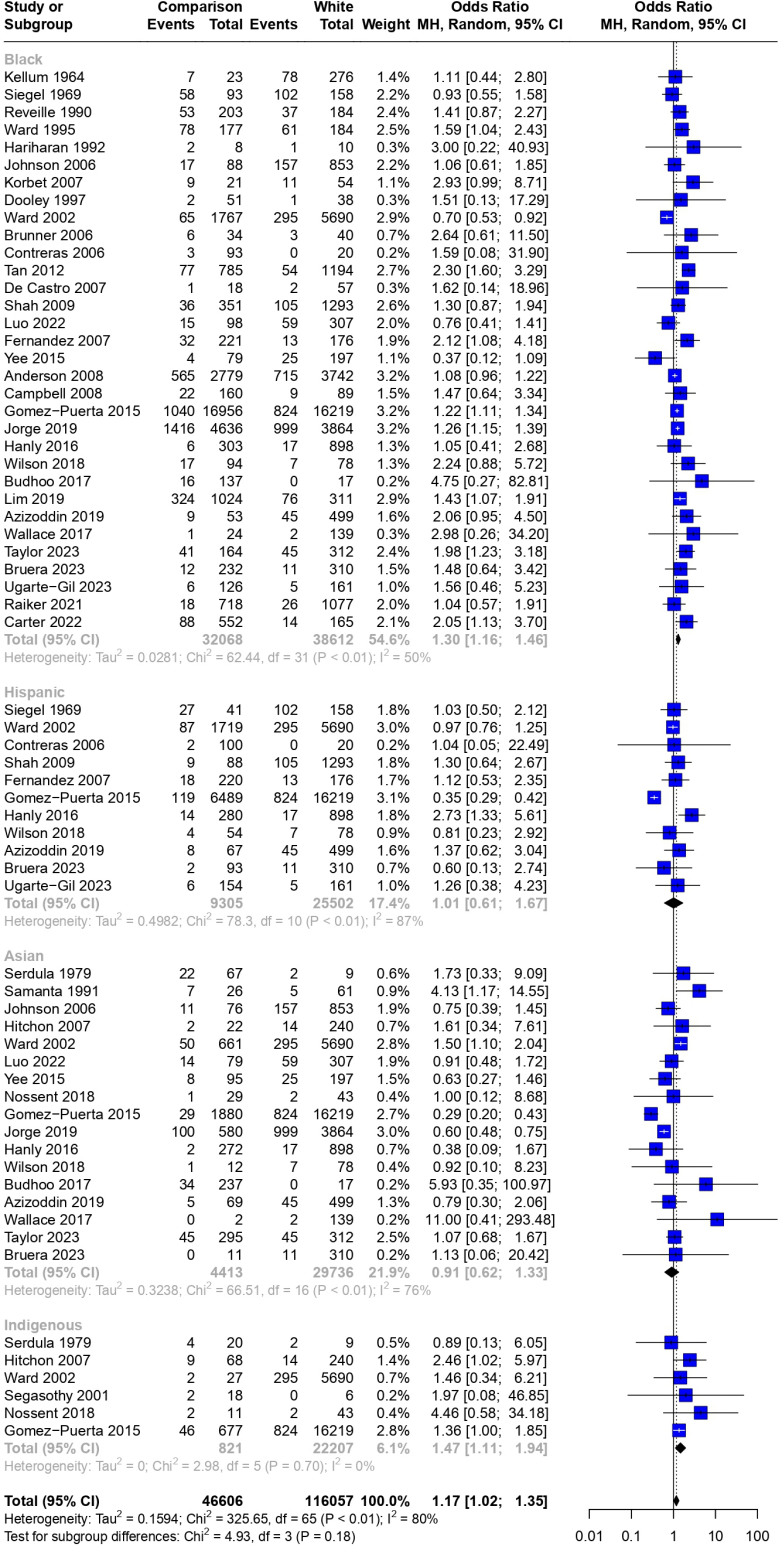
Association between race and ethnicity with mortality in patients with SLE. White race and ethnicity were used as the reference group for all comparisons. Weights are from random-effects analyses. Bars indicate 95% CIs. Heterogeneity between studies was assessed using I^2^ statistics.

Collectively, Asian, Black, Hispanic and Indigenous racial and ethnic groups were significantly associated with death compared with White racial and ethnic groups (OR 1.17 (95% CI 1.02 to 1.35)) (figure 2). However, heterogeneity was high among studies (I^2^=80%). The estimated pooled OR for mortality was higher in Black patients (OR 1.30 (95% CI 1.16 to 1.46), I^2^=50%) and Indigenous patients (OR 1.47 (95% CI 1.11 to 1.94), I^2^=0%) compared with White patients with SLE, whereas Asian or Hispanic patients showed no significant differences compared with White patients: OR 0.91 (95% CI 0.62 to 1.33) (I^2^=76%) and OR 1.01 (95% CI 0.61 to 1.67) (I^2^=87%), respectively.

Of the 17 studies that reported on Asian race and ethnicity, five specified between East Asian and four South Asian groups for their cohorts. In comparison with White race and ethnicity, no significant differences in mortality were found when these cohorts were analysed separately: East Asian OR 0.82 (95% CI 0.49 to 1.38) and South Asian OR 1.46 (95% CI 0.61 to 3.52) (online supplemental figure S1).

Meta-regression found no associations between study effect size, length of follow-up or mid-year point (online supplemental table S3). The association between race and ethnicity with mortality did not appear to change over time.

### Associations across countries of study

**Figure 3:**
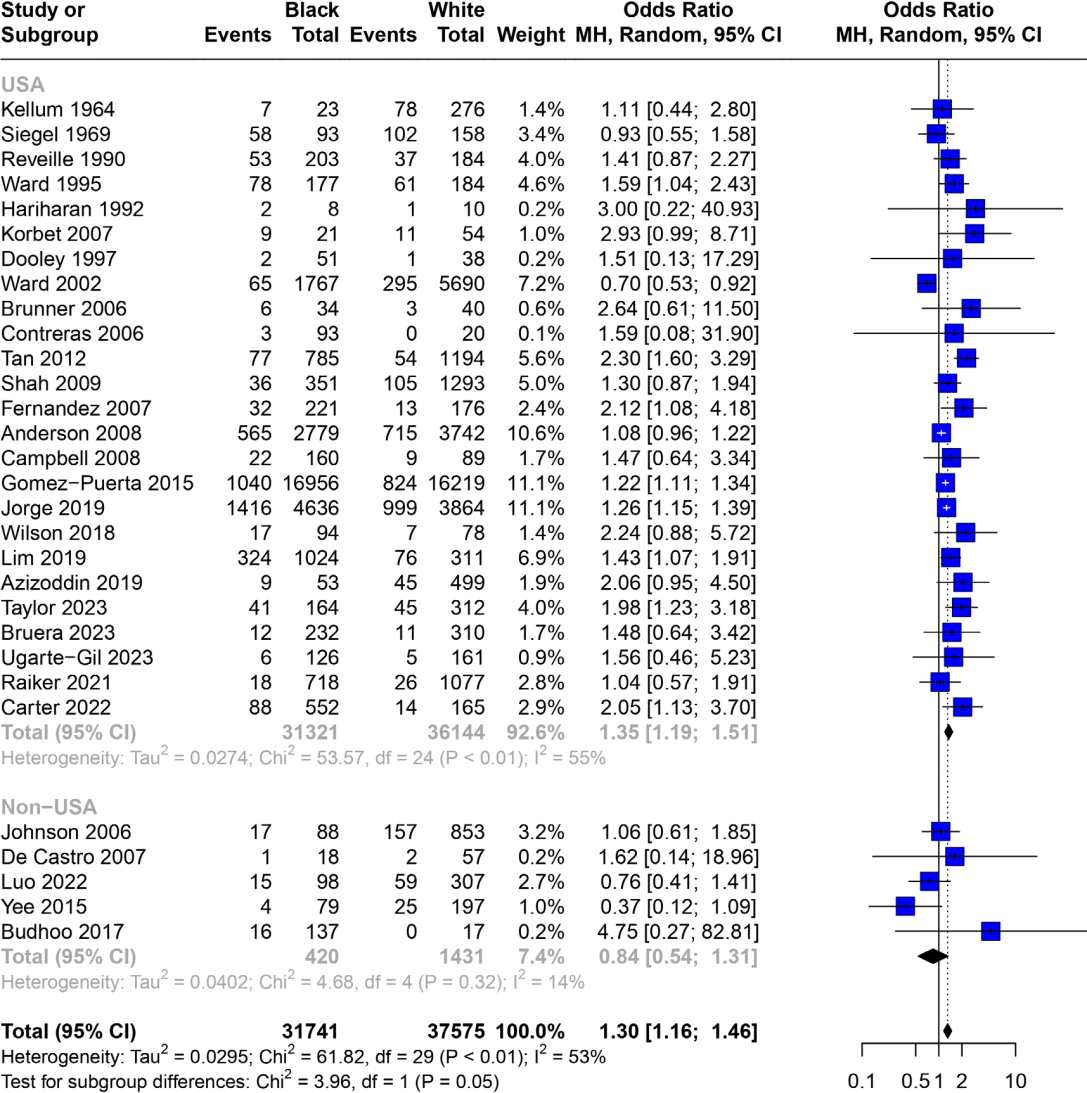
Subgroup analysis for the association of Black race and ethnicity with mortality in patients with SLE from USA-based and non-USA-based studies. White race and ethnicity were used as the reference for all comparisons. International studies were excluded. Weights are from random-effects analyses. Bars indicate 95% CIs. Heterogeneity between studies was assessed using I^2^ statistics.

The higher mortality observed in Black patients compared with White patients with SLE remained when restricted to the 25 USA-based studies (OR 1.35 (95% CI 1.19 to 1.51), figure 3).^18^,^42^ However, this difference in mortality was no longer seen on isolation of the five non-USA-based studies (OR 0.84 (95% CI 0.54 to 1.31)).^643^,^46^

Six studies described mortality for Indigenous and White patient groups with SLE (online supplemental figure S2).^2533 47^,^50^ Indigenous patients from both USA and non-USA sites had higher mortality than White patients (table 1). The non-USA-based studies had a higher and statistically significant OR, although with wide confidence intervals (OR 2.65 (95% CI 1.21 to 5.83)) compared with the USA group (OR 1.35 (95% CI 1.00 to 1.82)).

**Table 1:** Pooled OR of death in patients with SLE across race and ethnicity, categorised into USA-based and non-USA-based studies

Race and ethnicity	OverallOR (95% CI)	USA studiesOR (95% CI)	Non-USA studiesOR (95% CI)
White	Reference	Reference	Reference
Asian	0.91 (0.62 to 1.33)	0.80 (0.46 to 1.37)	1.06 (0.65 to 1.72)
Black	1.30 (1.16 to 1.46)	1.35 (1.19 to 1.51)	0.84 (0.54 to 1.31)
Hispanic	1.01 (0.61 to 1.67)	*	*
Indigenous	1.47 (1.11 to 1.94)	1.35 (1.00 to 1.82)	2.65 (1.21 to 5.83)

Expressed with 95% CIs. International studies were included in overall OR calculations only.

* No studies describing the mortality of White and Hispanic patients with SLE outside of the USA were deemed eligible.

The pooled OR of death in Asian patients compared with White patients with SLE showed no significant difference between USA-based and non-USA-based studies (table 1, online supplemental figure S3). No studies described the mortality of Hispanic and White patients outside of the USA.

### Associations across inception cohorts

Eight inception cohorts were included with mortality data for Asian, Black, Hispanic and White racial and ethnic groups^5 6 18 19 21 32 36 45^ (online supplemental figure S4). In these cohorts, no statistically significant risk of death was seen in the Black (OR 1.13 (95% CI 0.83 to 1.54)), Asian (OR 0.73 (95% CI 0.45 to 1.19)) or Hispanic groups (OR 1.68 (95% CI 0.64 to 4.36)) when compared with White patient groups.

Among all the included studies, 15 were deemed high risk of bias and 21 medium risk of bias (online supplemental table S4, S5). In a sensitivity analysis, whereby studies at high risk of bias were removed, Black patients with SLE continued to have significantly higher mortality than White patients (OR 1.28 (95% CI 1.13 to 1.46), online supplemental figure S5).

There was no indication of publication bias: Egger’s test (p=0.44); funnel plot provided in online supplemental figure S6.

## Discussion

Overall, people of Black or Indigenous race and ethnicity with SLE had a significantly higher mortality rate compared with those of White race and ethnicity, whereas the Hispanic and Asian groups showed no statistically significant differences in mortality compared with patients of White race and ethnicity. Despite differences in outcomes according to race and ethnicity, we noted geographical inconsistencies, especially between USA-based and non-USA-based studies. These findings support the hypothesis that race and ethnicity themselves may have no biological mechanism for the observed differences. Rather, it may be differences in access to care, socioeconomic deprivation and structural racism contained within race and ethnicity coding that are driving the associations.

Bernatsky *et al* reported a higher SMR for Black individuals (2.6) compared with White individuals with SLE (1.4) from a cohort of 3558 patients in the USA.^2^ In contrast to our study, they compared the mortality of patients with SLE against expected mortality from the general population; therefore, direct comparisons could not be made across races and ethnicities for those exclusively with SLE. Unfortunately, data from this study were not available for inclusion in our meta-analysis. Lee *et al* demonstrated equivalent SMRs between White and Asian patients with SLE (2.7 and 2.6, respectively) from a meta-analysis of European and Asian studies from 1990 to 2015.^51^ In comparison to our report, they pooled SMRs from single-ethnicity studies and did not describe the association of race and ethnicity with mortality in diverse populations as we have done.

The higher mortality observed in Black and Indigenous patients is likely to be driven by several diverse factors. Our findings may implicate a role for structural racism, which is increasingly recognised for its persistent and detrimental effects on patient outcomes.^52^ Notably, 70% of our included studies originated from the USA, indicating a strong underlying bias which limited the generalisability of our findings to other countries. Recent national data from the USA described higher mortality in the general Black and Indigenous American population compared with the White American population^13^ suggesting a wider systemic issue, which may be reflected in, but not limited to, patients with SLE. In contrast, recent population-level data from England showed higher age-standardised mortality rates in the White British population compared with the Black population.^53^ Indeed, if there were a higher risk of death in Black patients with SLE compared with White patients due to biological variation, we might expect this to hold true in England. However, there were no significant differences in mortality between racial and ethnic groups in two separate English cohorts.^6 45^ This finding suggests that the observed association between race and ethnicity with mortality in patients with SLE is more likely reflective of a wider population-level risk rather than a unique relationship within SLE. Many factors link structural racism to poor outcomes, including socioeconomic status, residential segregation, chronic stress, incarceration and discrimination in healthcare.^13^ Certain historic practices in the USA underscore this issue, for example, redlining of certain communities which inhibited the accumulation of generational wealth and social class migration, which likely impacted long-term health outcomes.

Limited data were reported on socioeconomic status and so we could not explore this variable in our models. However, discerning the individual impact of race, ethnicity and socioeconomic status on outcomes is difficult, as they are intertwined and arguably synergistic. Ward *et al* noted private medical insurance and median household income, not race or ethnicity, were significantly associated with mortality.^21^ Only five non-USA-based studies investigated the mortality of Black and White patients with SLE (from Canada, Brazil, South Africa and the UK),^643^,^46^ where no significant differences were seen. This likely reflected the variation in healthcare infrastructure and access, as four of these studies took place within universal healthcare systems^643^,^45^; whereas the USA hosts a combination of public and private insurance-based schemes. Unfortunately, studies of Indigenous patients with SLE were lacking for further analysis, with less numbers and detail compared with Black populations with SLE.

Differing health behaviours and literacy between races, ethnicities and cultures are also likely to contribute to poor outcomes. For example, poor medication concordance has been observed in young, Black and lower socioeconomic groups with SLE, with subsequently higher rates of emergency department visits and hospitalisation.^54^ This is compounded by the cost of healthcare in some countries, where lower income may prohibit engagement with therapies and services. Cultural and generational beliefs could also have a strong impact on patient engagement. For instance, historic racially unjust research practices, such as the Tuskegee Study, are commonly cited as the reason for the reduced participation of Black individuals in research^55^ and mistrust in healthcare.^56^

Alternatively, our results could be explained by a more severe SLE phenotype in Black and Indigenous racial and ethnic groups, which is poorly studied outside of the USA for Black patients and in general for Indigenous patients. Black patients are known to have an increased risk of lupus nephritis^5^ which has been associated with a high risk of death.^2^ Additionally, the presence of some high-risk genetic variants associated with kidney disease have been linked with recent African ancestry (eg, apolipoprotein L1).^57 58^ Black patients have also shown higher rates of organ damage compared with White patients in the USA,^59^ the accumulation of which is ultimately related to death^60^; however, damage could also be accrued via non-SLE or non-biological mechanisms.

A healthy migrant effect could also explain the lack of significant differences in the mortality of Asian and White groups demonstrated in our analysis. Black and Indigenous populations have a historic and deeply rooted role of persecution within the studied countries, whereas Asian and Hispanic migration occurred relatively more recently.^61^ No significant differences were seen when analysing USA-based versus non-USA-based studies for the Asian groups and a paucity of data outside of the USA was noted for Hispanic patients.

Our study had several strengths. To our knowledge, this was the first meta-analysis to examine mortality across multiple races and ethnicities in patients with SLE. Our search was comprehensive and incorporated many studies over a long period, providing a broad lens to this important area of health inequity. Multiple sensitivity analyses and meta-regression were used to further explore findings.

By its nature, this meta-analysis was subject to limitations. First, we relied on published data and could not consistently find detailed descriptions of age, gender, race and ethnicity, comorbidities, organs involved, socioeconomic status or numbers lost to follow-up. Therefore, we were unable to adjust for these additional variables and we estimated the effect of race and ethnicity on mortality using the total number of deaths reported. Second, many studies grouped sets of ethnicities together, for example, Asian, which introduced substantial heterogeneity given the broad nature of this group label. We attempted to address this by separately analysing outcomes for South and East Asian patients where defined. Similarly, we were unable to gain further detail into the Black population, for example, Black African, Black Caribbean, African American patients, all of whom have a different incidence of SLE^1^ and likely experience different environmental stresses. Third, patients who identified as Hispanic may have been categorised into White or Black racial and ethnic groups in some studies due to the ambiguity between race and ethnicity. Fourth, the inclusion of large national registries from the USA may have led to inadvertent duplication of some patients from included local cohorts. There was also the potential for selection bias within prospective studies, as low participation seen in some racial and ethnic minority groups may have led to under-representation.^62^ Finally, the involved studies ranged from 1949 to 2021, encompassing inherent time-related differences in the classification of SLE, treatments, patient and physician attitudes towards SLE and differing disparities over time.

Our findings promote caution in the use of race and ethnicity-derived practices due to the potential for misinterpretation and conflation of correlation for causation. The importance of highlighting racial and ethnic disparities lies in exposing injustice, increasing transparency, questioning physician-led bias and propagating further research. This is particularly important in racially and ethnically diverse populations to ensure equitable distribution of resources and care, especially if socioeconomic factors,^63^ and not race and ethnicity per se, are key determinants of mortality. Further work should involve large national SLE survival studies outside of the USA and culturally sensitive qualitative research to understand and improve engagement with SLE communities.

## Conclusion

We identified higher mortality for Black and Indigenous patients with SLE compared with White patients; however, we noticed inconsistencies between countries of study. This was especially true for Black patients, who had a higher mortality compared with White patients in the USA, but not in studies from outside of the USA. We highlighted a scarcity of data from other countries and propose that race and ethnicity should be routinely reported in SLE studies to tackle this. Our report emphasised the need for re-examination and edification of the current SLE literature with regard to race, ethnicity and outcomes.

## supplementary material

10.1136/lupus-2024-001383 online supplemental file 1

## Data Availability

All data relevant to the study are included in the article or uploaded as supplementary information.
