# Supplementary material for: Association of race and ethnicity with mortality in adults with SLE: a systematic literature review and meta-analysis
Source: Lupus Sci Med. 2025 Feb 11;12(1):e001383. doi: 10.1136/lupus-2024-001383 (PMC11815449; doi:10.1136/lupus-2024-001383)
Supplement: online supplemental file 1 [file lupus-12-1-s001.pdf]

Supplementary

Supplementary figure S1. Mortality by race and ethnicity in patients with SLE including East and South Asian subgroups. White race and ethnicity were used as the reference for all comparisons. Weights are from random-effects analyses. Bars indicate 95% confidence intervals (CI).

Study or Subgroup	Comparison Events	Comparison Total	White Events	White Total	Weight	Odds Ratio MH, Random, 95% CI
Black						
Kellum 1964	7	23	78	276	1.5%	1.11 [0.44; 2.80]
Siegel 1969	58	93	102	158	2.6%	0.93 [0.55; 1.58]
Reveille 1990	53	203	37	184	2.8%	1.41 [0.87; 2.27]
Ward 1995	78	177	61	184	3.0%	1.59 [1.04; 2.43]
Hariharan 1992	2	8	1	10	0.3%	3.00 [0.22; 40.93]
Johnson 2006	17	88	157	853	2.5%	1.06 [0.61; 1.85]
Korbet 2007	9	21	11	54	1.2%	2.93 [0.99; 8.71]
Dooley 1997	2	51	1	38	0.3%	1.51 [0.13; 17.29]
Ward 2002	65	1767	295	5690	3.6%	0.70 [0.53; 0.92]
Brunner 2006	6	34	3	40	0.8%	2.64 [0.61; 11.50]
Contreras 2006	3	93	0	20	0.2%	1.59 [0.08; 31.90]
Tan 2012	77	785	54	1194	3.2%	2.30 [1.60; 3.29]
De Castro 2007	1	18	2	57	0.3%	1.62 [0.14; 18.96]
Shah 2009	36	351	105	1293	3.1%	1.30 [0.87; 1.94]
Luo 2022	15	98	59	307	2.3%	0.76 [0.41; 1.41]
Fernandez 2007	32	221	13	176	2.1%	2.12 [1.08; 4.18]
Yee 2015	4	79	25	197	1.2%	0.37 [0.12; 1.09]
Anderson 2008	565	2779	715	3742	4.0%	1.08 [0.96; 1.22]
Campbell 2008	22	160	9	89	1.7%	1.47 [0.64; 3.34]
Gomez−Puerta 2015	1040	16956	824	16219	4.0%	1.22 [1.11; 1.34]
Jorge 2019	1416	4636	999	3864	4.0%	1.26 [1.15; 1.39]
Hanly 2016	6	303	17	898	1.5%	1.05 [0.41; 2.68]
Wilson 2018	17	94	7	78	1.5%	2.24 [0.88; 5.72]
Budhoo 2017	16	137	0	17	0.2%	4.75 [0.27; 82.81]
Lim 2019	324	1024	76	311	3.5%	1.43 [1.07; 1.91]
Azizoddin 2019	9	53	45	499	1.8%	2.06 [0.95; 4.50]
Wallace 2017	1	24	2	139	0.3%	2.98 [0.26; 34.20]
Taylor 2023	41	164	45	312	2.8%	1.98 [1.23; 3.18]
Bruera 2023	12	232	11	310	1.7%	1.48 [0.64; 3.42]
Ugarte−Gil 2023	6	126	5	161	1.0%	1.56 [0.46; 5.23]
Carter 2022	88	552	14	165	2.4%	2.05 [1.13; 3.70]
Raiker 2021	18	718	26	1077	2.3%	1.04 [0.57; 1.91]
Total (95% CI)		32068		38612	63.7%	1.30 [1.16; 1.46]
Heterogeneity: $Tau^2 = 0.0281$; $Chi^2 = 62.44$, df = 31 ($P < 0.01$); $I^2 = 50\%$						
Hispanic						
Siegel 1969	27	41	102	158	2.0%	1.03 [0.50; 2.12]
Ward 2002	87	1719	295	5690	3.7%	0.97 [0.76; 1.25]
Contreras 2006	2	100	0	20	0.2%	1.04 [0.05; 22.49]
Shah 2009	9	88	105	1293	2.0%	1.30 [0.64; 2.67]
Fernandez 2007	18	220	13	176	1.9%	1.12 [0.53; 2.35]
Gomez−Puerta 2015	119	6489	824	16219	3.8%	0.35 [0.29; 0.42]
Hanly 2016	14	280	17	898	2.0%	2.73 [1.33; 5.61]
Wilson 2018	4	54	7	78	0.9%	0.81 [0.23; 2.92]
Azizoddin 2019	8	67	45	499	1.8%	1.37 [0.62; 3.04]
Bruera 2023	2	93	11	310	0.7%	0.60 [0.13; 2.74]
Ugarte−Gil 2023	6	154	5	161	1.0%	1.26 [0.38; 4.23]
Total (95% CI)		9305		25502	20.0%	1.01 [0.61; 1.67]
Heterogeneity: $Tau^2 = 0.4982$; $Chi^2 = 78.3$, df = 10 ($P < 0.01$); $I^2 = 87\%$						
E Asian						
Serdula 1979	22	67	2	9	0.6%	1.73 [0.33; 9.09]
Johnson 2006	11	76	157	853	2.2%	0.75 [0.39; 1.45]
Hitchon 2007	2	22	14	240	0.7%	1.61 [0.34; 7.61]
Luo 2022	2	24	59	307	0.7%	0.38 [0.09; 1.67]
Yee 2015	1	9	25	197	0.4%	0.86 [0.10; 7.17]
Total (95% CI)		198		1606	4.6%	0.82 [0.49; 1.38]
Heterogeneity: $Tau^2 = 0$; $Chi^2 = 2.64$, df = 4 ($P = 0.62$); $I^2 = 0\%$						
Indigenous						
Serdula 1979	4	20	2	9	0.5%	0.89 [0.13; 6.05]
Hitchon 2007	9	68	14	240	1.6%	2.46 [1.02; 5.97]
Ward 2002	2	27	295	5690	0.8%	1.46 [0.34; 6.21]
Segasothy 2001	2	18	0	6	0.2%	1.97 [0.08; 46.85]
Nossent 2018	2	11	2	43	0.4%	4.46 [0.58; 34.18]
Gomez−Puerta 2015	46	677	824	16219	3.4%	1.36 [1.00; 1.85]
Total (95% CI)		821		22207	6.9%	1.47 [1.11; 1.94]
Heterogeneity: $Tau^2 = 0$; $Chi^2 = 2.98$, df = 5 ($P = 0.70$); $I^2 = 0\%$						
S Asian						
Samanta 1991	7	26	5	61	1.0%	4.13 [1.17; 14.55]
Luo 2022	12	55	59	307	2.0%	1.17 [0.58; 2.36]
Yee 2015	7	86	25	197	1.6%	0.61 [0.25; 1.47]
Budhoo 2017	34	237	0	17	0.2%	5.93 [0.35; 100.97]
Total (95% CI)		404		582	4.8%	1.46 [0.61; 3.52]
Heterogeneity: $Tau^2 = 0.4275$; $Chi^2 = 7.2$, df = 3 ($P = 0.07$); $I^2 = 58\%$						
**Total (95% CI)**		**42796**		**88509**	**100.0%**	**1.25 [1.08; 1.44]**
Heterogeneity: $Tau^2 = 0.1265$; $Chi^2 = 240.15$, df = 57 ($P < 0.01$); $I^2 = 76\%$						
Test for subgroup differences: $Chi^2 = 4.77$, df = 4 ($P = 0.31$)						

Odds Ratio MH, Random, 95% CI

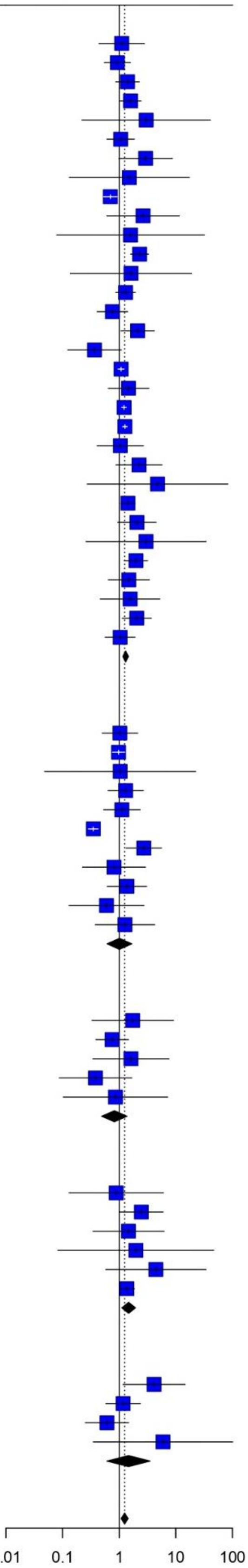

Supplementary figure S2. Subgroup analysis for the association of Indigenous race and ethnicity with mortality in patients with SLE from USA based and non-USA-based studies. Weights are from random-effects analyses. White race and ethnicity were used as the reference for all comparisons. Bars indicate 95% confidence intervals (CI). Heterogeneity between studies was assessed using I^2 statistics.

Study or Subgroup	Indigenous Events	Indigenous Total	White Events	White Total	Weight	Odds Ratio MH, Random, 95% CI
USA						
Serdula 1979	4	20	2	9	2.1%	0.89 [0.13; 6.05]
Ward 2002	2	27	295	5690	3.7%	1.46 [0.34; 6.21]
Gomez−Puerta 2015	46	677	824	16219	81.7%	1.36 [1.00; 1.85]
Total (95% CI)		724		21918	87.5%	1.35 [1.00; 1.82]
Heterogeneity: $Tau^2 = 0$; $Chi^2 = 0.2$, df = 2 (P = 0.91); $I^2 = 0\%$						
Non−USA						
Hitchon 2007	9	68	14	240	9.9%	2.46 [1.02; 5.97]
Segasothy 2001	2	18	0	6	0.8%	1.97 [0.08; 46.85]
Nossent 2018	2	11	2	43	1.9%	4.46 [0.58; 34.18]
Total (95% CI)		97		289	12.5%	2.65 [1.21; 5.83]
Heterogeneity: $Tau^2 = 0$; $Chi^2 = 0.31$, df = 2 (P = 0.86); $I^2 = 0\%$						
**Total (95% CI)**		**821**		**22207**	**100.0%**	**1.47 [1.11; 1.94]**
Heterogeneity: $Tau^2 = 0$; $Chi^2 = 2.98$, df = 5 (P = 0.70); $I^2 = 0\%$						
Test for subgroup differences: $Chi^2 = 2.47$, df = 1 (P = 0.12)						

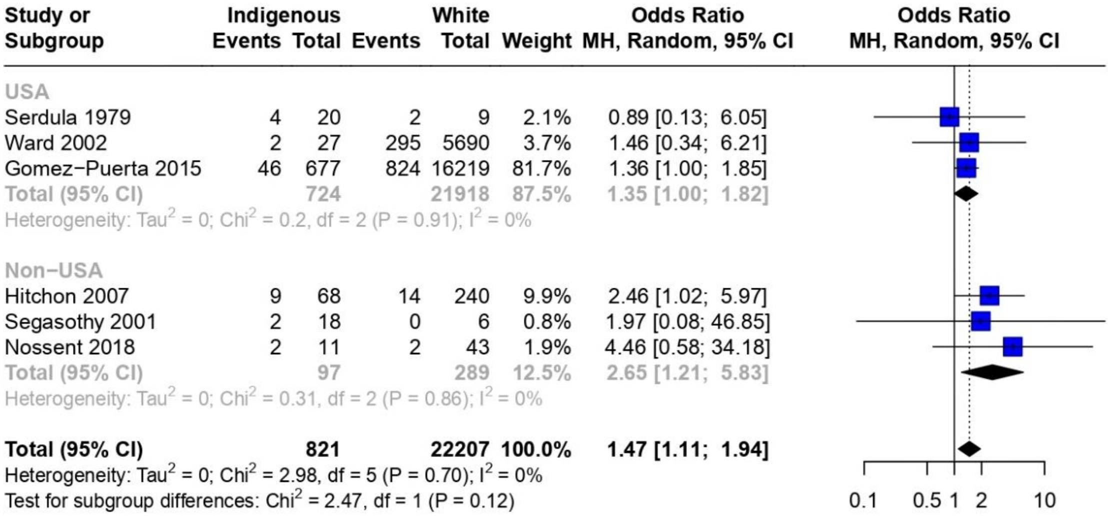

Supplementary figure S3. Subgroup analysis for the association of Asian race and ethnicity with mortality in patients with SLE from USA based and non-USA-based studies. Weights are from random-effects analyses. White race and ethnicity were used as the reference for all comparisons. International studies were excluded. Bars indicate 95% confidence intervals (CI). Heterogeneity between studies was assessed using I^2 statistics.

Study or Subgroup	Asian Events	Asian Total	White Events	White Total	Weight	Odds Ratio MH, Random, 95% CI
USA						
Serdula 1979	22	67	2	9	3.8%	1.73 [0.33; 9.09]
Ward 2002	50	661	295	5690	11.5%	1.50 [1.10; 2.04]
Gomez−Puerta 2015	29	1880	824	16219	11.1%	0.29 [0.20; 0.43]
Jorge 2019	100	580	999	3864	11.9%	0.60 [0.48; 0.75]
Wilson 2018	1	12	7	78	2.5%	0.92 [0.10; 8.23]
Azizoddin 2019	5	69	45	499	7.1%	0.79 [0.30; 2.06]
Taylor 2023	45	295	45	312	10.6%	1.07 [0.68; 1.67]
Bruera 2023	0	11	11	310	1.6%	1.13 [0.06; 20.42]
Total (95% CI)		3575		26981	60.0%	0.80 [0.46; 1.37]
Heterogeneity: $Tau^2 = 0.3840$; $Chi^2 = 52.45$, df = 7 ($P < 0.01$); $I^2 = 87\%$						
Non−USA						
Samanta 1991	7	26	5	61	5.4%	4.13 [1.17; 14.55]
Johnson 2006	11	76	157	853	9.1%	0.75 [0.39; 1.45]
Hitchon 2007	2	22	14	240	4.2%	1.61 [0.34; 7.61]
Luo 2022	14	79	59	307	9.2%	0.91 [0.48; 1.72]
Yee 2015	8	95	25	197	7.9%	0.63 [0.27; 1.46]
Nossent 2018	1	29	2	43	2.6%	1.00 [0.12; 8.68]
Budhoo 2017	34	237	0	17	1.6%	5.93 [0.35; 100.97]
Total (95% CI)		564		1718	40.0%	1.06 [0.65; 1.72]
Heterogeneity: $Tau^2 = 0.1232$; $Chi^2 = 8.69$, df = 6 ($P = 0.19$); $I^2 = 31\%$						
**Total (95% CI)**		**4139**		**28699**	**100.0%**	**0.92 [0.62; 1.35]**
Heterogeneity: $Tau^2 = 0.3196$; $Chi^2 = 63.03$, df = 14 ($P < 0.01$); $I^2 = 78\%$						
Test for subgroup differences: $Chi^2 = 0.58$, df = 1 ($P = 0.44$)						

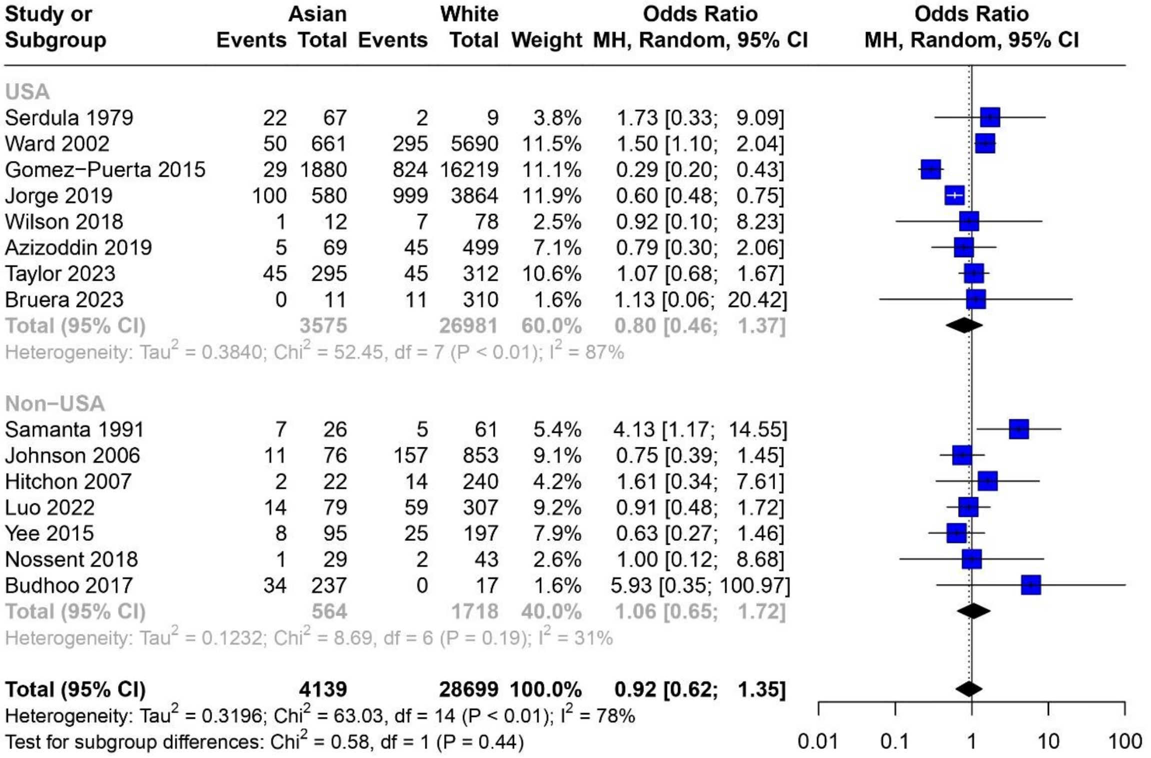

Supplementary figure S4. Association between race and ethnicity with mortality in patients with SLE from inception cohorts. Weights are from random-effects analyses. White race and ethnicity were used as the reference for all comparisons. Bars indicate 95% confidence intervals (CI). Heterogeneity between studies was assessed using I^2 statistics.

Study or Subgroup	Comparison Events	Comparison Total	White Events	White Total	Weight	Odds Ratio MH, Random, 95% CI
**Black**						
Kellum 1964	7	23	78	276	5.9%	1.11 [0.44; 2.80]
Siegel 1969	58	93	102	158	10.7%	0.93 [0.55; 1.58]
Ward 1995	78	177	61	184	12.6%	1.59 [1.04; 2.43]
Luo 2022	15	98	59	307	9.4%	0.76 [0.41; 1.41]
Yee 2015	4	79	25	197	4.7%	0.37 [0.12; 1.09]
Campbell 2008	22	160	9	89	6.8%	1.47 [0.64; 3.34]
Hanly 2016	6	303	17	898	5.7%	1.05 [0.41; 2.68]
Lim 2019	83	248	16	77	9.6%	1.87 [1.02; 3.43]
**Total (95% CI)**		**1181**		**2186**	**65.4%**	**1.13 [0.83; 1.54]**
Heterogeneity: $Tau^2 = 0.0759$; $Chi^2 = 11.57$, df = 7 (P = 0.12); $I^2 = 40\%$						
**Hispanic**						
Siegel 1969	27	41	102	158	8.0%	1.03 [0.50; 2.12]
Hanly 2016	14	280	17	898	8.0%	2.73 [1.33; 5.61]
**Total (95% CI)**		**321**		**1056**	**16.0%**	**1.68 [0.64; 4.36]**
Heterogeneity: $Tau^2 = 0.3409$; $Chi^2 = 3.52$, df = 1 (P = 0.06); $I^2 = 72\%$						
**Asian**						
Luo 2022	14	79	59	307	9.0%	0.91 [0.48; 1.72]
Yee 2015	8	95	25	197	6.7%	0.63 [0.27; 1.46]
Hanly 2016	2	272	17	898	2.9%	0.38 [0.09; 1.67]
**Total (95% CI)**		**446**		**1402**	**18.6%**	**0.73 [0.45; 1.19]**
Heterogeneity: $Tau^2 = 0$; $Chi^2 = 1.29$, df = 2 (P = 0.53); $I^2 = 0\%$						
**Total (95% CI)**		**1948**		**4644**	**100.0%**	**1.09 [0.83; 1.43]**
Heterogeneity: $Tau^2 = 0.1064$; $Chi^2 = 21.92$, df = 12 (P = 0.04); $I^2 = 45\%$						
Test for subgroup differences: $Chi^2 = 3.24$, df = 2 (P = 0.20)						

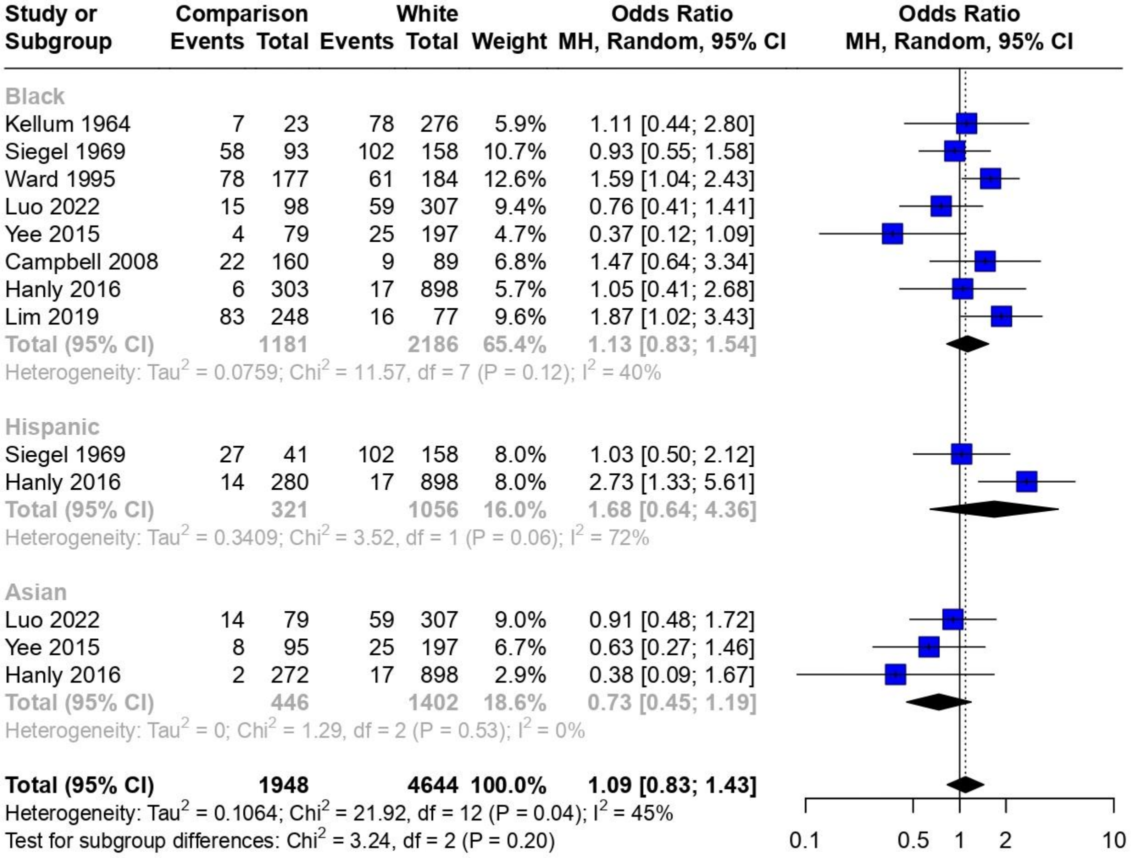

Supplementary figure S5. Association between Black race and ethnicity with mortality in patients with SLE excluding studies with high risk of bias. Weights are from random-effects analyses. White race and ethnicity were used as the reference for all comparisons. Bars indicate 95% confidence intervals (CI). Heterogeneity between studies was assessed using I^2 statistics.

Study	Black Events	Black Total	White Events	White Total	Weight	Odds Ratio MH, Random, 95% CI
Reveille 1990	53	203	37	184	4.9%	1.41 [0.87; 2.27]
Ward 1995	78	177	61	184	5.6%	1.59 [1.04; 2.43]
Johnson 2006	17	88	157	853	3.9%	1.06 [0.61; 1.85]
Korbet 2007	9	21	11	54	1.3%	2.93 [0.99; 8.71]
Ward 2002	65	1767	295	5690	8.9%	0.70 [0.53; 0.92]
Tan 2012	77	785	54	1194	6.9%	2.30 [1.60; 3.29]
Shah 2009	36	351	105	1293	6.1%	1.30 [0.87; 1.94]
Fernandez 2007	32	221	13	176	2.9%	2.12 [1.08; 4.18]
Yee 2015	4	79	25	197	1.3%	0.37 [0.12; 1.09]
Anderson 2008	565	2779	715	3742	13.1%	1.08 [0.96; 1.22]
Campbell 2008	22	160	9	89	2.1%	1.47 [0.64; 3.34]
Gomez−Puerta 2015	1040	16956	824	16219	13.8%	1.22 [1.11; 1.34]
Jorge 2019	1416	4636	999	3864	13.8%	1.26 [1.15; 1.39]
Hanly 2016	6	303	17	898	1.7%	1.05 [0.41; 2.68]
Wilson 2018	17	94	7	78	1.7%	2.24 [0.88; 5.72]
Budhoo 2017	16	137	0	17	0.2%	4.75 [0.27; 82.81]
Lim 2019	324	1024	76	311	8.5%	1.43 [1.07; 1.91]
Wallace 2017	1	24	2	139	0.3%	2.98 [0.26; 34.20]
Bruera 2023	12	232	11	310	2.0%	1.48 [0.64; 3.42]
Ugarte−Gil 2023	6	126	5	161	1.1%	1.56 [0.46; 5.23]
**Total (95% CI)**		**30163**		**35653**	**100.0%**	**1.28 [1.13; 1.46]**

Heterogeneity: $Tau^2 = 0.0289$; $Chi^2 = 48.63$, df = 19 ($P < 0.01$); $I^2 = 61\%$

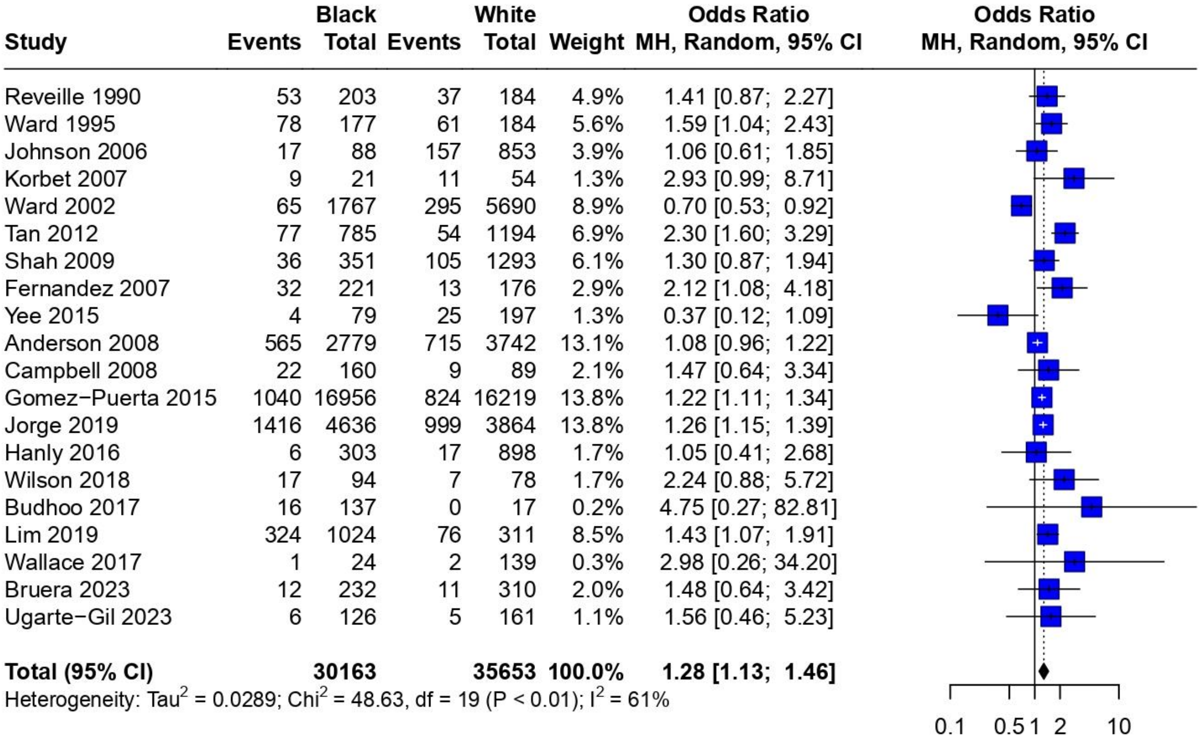

Supplementary figure S6. Funnel plot for publication bias.

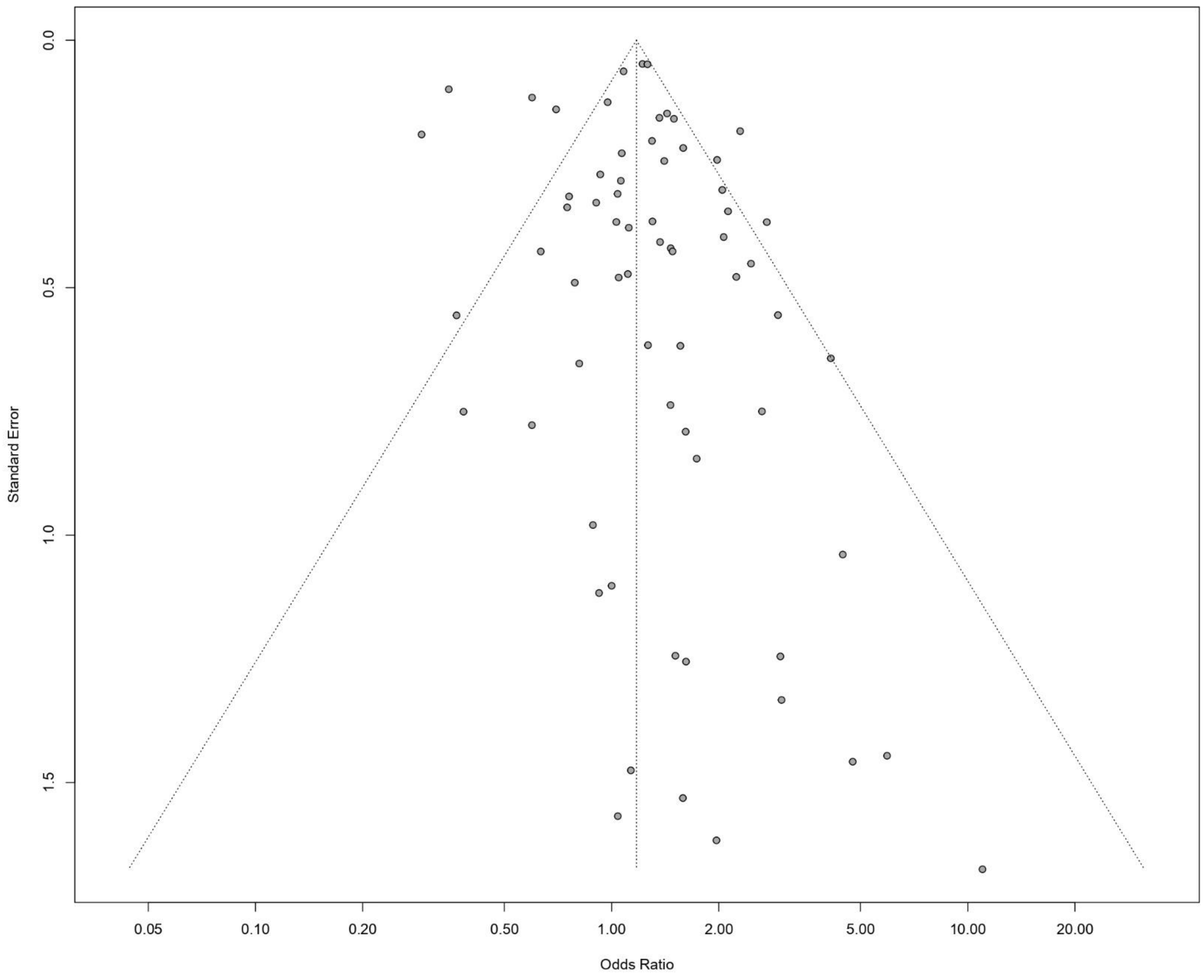

Supplementary table S1. Literature search terms.

1 systemic lupus erythematosus.mp. [mp=ti, ab, hw, tn, ot, dm, mf, dv, kf, fx, dq, bt, nm, ox, px, rx, ui, sy, ux, mx]

2 SLE.mp. [mp=ti, ab, hw, tn, ot, dm, mf, dv, kf, fx, dq, bt, nm, ox, px, rx, ui, sy, ux, mx]

3 systemic lupus.mp. [mp=ti, ab, hw, tn, ot, dm, mf, dv, kf, fx, dq, bt, nm, ox, px, rx, ui, sy, ux, mx]

4 lupus.mp. [mp=ti, ab, hw, tn, ot, dm, mf, dv, kf, fx, dq, bt, nm, ox, px, rx, ui, sy, ux, mx]

5 lupus nephritis.mp. [mp=ti, ab, hw, tn, ot, dm, mf, dv, kf, fx, dq, bt, nm, ox, px, rx, ui, sy, ux, mx]

6 SLE nephritis.mp. [mp=ti, ab, hw, tn, ot, dm, mf, dv, kf, fx, dq, bt, nm, ox, px, rx, ui, sy, ux, mx]

7 lupus glomerulonephritis.mp. [mp=ti, ab, hw, tn, ot, dm, mf, dv, kf, fx, dq, bt, nm, ox, px, rx, ui, sy, ux, mx]

8 SLE glomerulonephritis.mp. [mp=ti, ab, hw, tn, ot, dm, mf, dv, kf, fx, dq, bt, nm, ox, px, rx, ui, sy, ux, mx]

9 exp Ethnicity/

10 ethnicity.mp. [mp=ti, ab, hw, tn, ot, dm, mf, dv, kf, fx, dq, bt, nm, ox, px, rx, ui, sy, ux, mx]

11 Black ethnicity.mp. [mp=ti, ab, hw, tn, ot, dm, mf, dv, kf, fx, dq, bt, nm, ox, px, rx, ui, sy, ux, mx]

12 population group?.mp. [mp=ti, ab, hw, tn, ot, dm, mf, dv, kf, fx, dq, bt, nm, ox, px, rx, ui, sy, ux, mx]

13 exp racial groups/ or exp blacks/ or exp american native continental ancestry group/ or exp asians/ or exp whites/ or exp "native hawaiian or other pacific islander"/

14 race.mp. [mp=ti, ab, hw, tn, ot, dm, mf, dv, kf, fx, dq, bt, nm, ox, px, rx, ui, sy, ux, mx]

15 racial.mp. [mp=ti, ab, hw, tn, ot, dm, mf, dv, kf, fx, dq, bt, nm, ox, px, rx, ui, sy, ux, mx]

16 exp blacks/ or exp african americans/

17 black adult*.mp. [mp=ti, ab, hw, tn, ot, dm, mf, dv, kf, fx, dq, bt, nm, ox, px, rx, ui, sy, ux, mx]

18 exp african americans/ or exp vulnerable populations/

19 African American?.mp. [mp=ti, ab, hw, tn, ot, dm, mf, dv, kf, fx, dq, bt, nm, ox, px, rx, ui, sy, ux, mx]

20 Afro-caribbean?.mp. [mp=ti, ab, hw, tn, ot, dm, mf, dv, kf, fx, dq, bt, nm, ox, px, rx, ui, sy, ux, mx]

21 African.mp. [mp=ti, ab, hw, tn, ot, dm, mf, dv, kf, fx, dq, bt, nm, ox, px, rx, ui, sy, ux, mx]

22 exp Minority Groups/

23 minorit*.mp. [mp=ti, ab, hw, tn, ot, dm, mf, dv, kf, fx, dq, bt, nm, ox, px, rx, ui, sy, ux, mx]

24 exp "Ethnic and Racial Minorities"/

25 exp Mortality/

26 mortality.mp. [mp=ti, ab, hw, tn, ot, dm, mf, dv, kf, fx, dq, bt, nm, ox, px, rx, ui, sy, ux, mx]

27 exp hospital* mortality/ or exp mortality, premature/ or exp survival rate/

28 hospital mortality.mp. [mp=ti, ab, hw, tn, ot, dm, mf, dv, kf, fx, dq, bt, nm, ox, px, rx, ui, sy, ux, mx]

29 exp Survival/

30	survival*.mp. [mp=ti, ab, hw, tn, ot, dm, mf, dv, kf, fx, dq, bt, nm, ox, px, rx, ui, sy, ux, mx]
31	fatal outcome*.mp. [mp=ti, ab, hw, tn, ot, dm, mf, dv, kf, fx, dq, bt, nm, ox, px, rx, ui, sy, ux, mx]
32	exp fatal outcome/
33	exp health inequities/ or exp health status disparities/
34	health* disparit*.mp. [mp=ti, ab, hw, tn, ot, dm, mf, dv, kf, fx, dq, bt, nm, ox, px, rx, ui, sy, ux, mx]
35	health* inequ*.mp. [mp=ti, ab, hw, tn, ot, dm, mf, dv, kf, fx, dq, bt, nm, ox, px, rx, ui, sy, ux, mx]
36	exp Death/
37	death*.mp. [mp=ti, ab, hw, tn, ot, dm, mf, dv, kf, fx, dq, bt, nm, ox, px, rx, ui, sy, ux, mx]
38	exp treatment outcome/ or exp disease-free survival/
39	outcome*.mp. [mp=ti, ab, hw, tn, ot, dm, mf, dv, kf, fx, dq, bt, nm, ox, px, rx, ui, sy, ux, mx]
40	exp adverse outcome/
41	1 or 2 or 3 or 4 or 5 or 6 or 7 or 8
42	9 or 10 or 11 or 12 or 13 or 14 or 15 or 16 or 17 or 18 or 19 or 20 or 21 or 22 or 23 or 24
43	25 or 26 or 27 or 28 or 29 or 30 or 31 or 32 or 33 or 34 or 35 or 36 or 37 or 38 or 39 or 40
44	41 and 42 and 43
45	limit 44 to english language

Supplementary table S2. Study characteristics (- denotes missing data)

Study	Study design	Study country	Study period	Registry or database	Racial and Ethnic groups (W, White; B, Black; H, Hispanic; A, Asian; I, Indigenous)	Total number (n)	Female (SD)	White race and ethnicity (n)	White race and ethnicity deaths (n)	Black race and ethnicity (n)	Black race and ethnicity deaths (n)	Hispanic race and ethnicity (n)	Hispanic race and ethnicity deaths (n)	Asian race and ethnicity (n)	Asian race and ethnicity deaths (n)	Indigenous race and ethnicity (n)	Indigenous race and ethnicity deaths (n)
**Nossent et al (2018) (49)**	Observational: retrospective cohort	Australia	1997 - 2017		W/A/I	83	87.9% (10.5)	43	2	-	-	-	-	29	1	11	2
**Segasothy et al (2001) (48)**	Observational: retrospective cohort	Australia	1990 - 1999		W/I	24	83.3% (0)	6	0	-	-	-	-	-	-	18	2
**De Castro et al (2007) (44)**	Observational: retrospective cohort	Brazil	1988 - 2003		W/B	75	88.4% (8.4)	57	2	18	1	-	-	-	-	-	-
**Hitchon et al (2007) (50)**	Observational: retrospective cohort	Canada	1980 - 2001		W/A/I	330	89.3% (1.5)	240	14	-	-	-	-	22	2	68	9
**Johnson et al (2006) (43)**	Observational: prospective cohort	Canada	1970 - 2002		W/B/A	1017	88.8% (4.8)	853	157	88	17	-	-	76	11	-	-
**Hanly et al (2016) (5)**	Observational: prospective cohort	International	1999 - 2012	X	W/B/H/A	1753	-	898	17	303	6	280	14	272	2	-	-
**Wallace et al (2017) (64)**	Randomized controlled trial	International	2011 - 2014		W/B/A	165	-	139	2	24	1	-	-	2	0	-	-
**Budhoo et al (2017) (46)**	Observational: retrospective cohort	South Africa	2003 - 2012		W/B/A	391	92.3% (1.6)	17	0	137	16	-	-	237	34	-	-
**Luo et al (2022) (6)**	Observational: retrospective cohort	UK	1979 - 2019		W/B/A	484	-	307	59	98	15	-	-	79	14	-	-
**Samanta et al (1991) (65)**	Observational: retrospective cohort	UK	1979 - 1988		W/A	87	78.0% (12.7)	61	5	-	-	-	-	26	7	-	-
**Yee et al (2015) (45)**	Observational: prospective cohort	UK	1989 - 2010		W/B/A	371	-	197	25	79	4	-	-	95	8	-	-
**Anderson et al (2008) (31)**	Observational: retrospective cohort	USA	1996 - 2003	X	W/B	6521	87.8% (0.1)	3742	715	2779	565	-	-	-	-	-	-
**Azizoddin et al (2019) (37)**	Observational: prospective cohort	USA	2007 - 2015	X	W/B/H/A	688	-	499	45	53	9	67	8	69	5	-	-
**Bruera et al (2023) (39)**	Observational: retrospective cohort	USA	2020	X	W/B/H/A	646	-	310	11	232	12	93	2	11	0	-	-
**Brunner et al (2006) (26)**	Observational: retrospective cohort	USA	1981 - 2002		W/B	74	-	40	3	34	6	-	-	-	-	-	-
**Campbell et al (2008) (32)**	Observational: prospective cohort	USA	1997 - 2004	X	W/B	249	-	89	9	160	22	-	-	-	-	-	-

Study	Study design	Study country	Study period	Registry or database	Racial and Ethnic groups (W, White; B, Black; H, Hispanic; A, Asian; I, Indigenous)	Total number (n)	Female (SD)	White race and ethnicity (n)	White race and ethnicity deaths (n)	Black race and ethnicity (n)	Black race and ethnicity deaths (n)	Hispanic race and ethnicity (n)	Hispanic race and ethnicity deaths (n)	Asian race and ethnicity (n)	Asian race and ethnicity deaths (n)	Indigenous race and ethnicity (n)	Indigenous race and ethnicity deaths (n)
**Carter et al (2022) (42)**	Observational: retrospective cohort	USA	-		W/B	717	-	165	14	552	88	-	-	-	-	-	-
**Contreras et al (2006) (27)**	Observational: retrospective cohort	USA	1983 - 2003		W/B/H	213	87.7% (2.3)	20	0	93	3	100	2	-	-	-	-
**Dooley et al (1997) (24)**	Observational: prospective cohort	USA	1985 - 1995	X	W/B	89	85.2% (1.5)	38	1	51	2	-	-	-	-	-	-
**Gomez-Puerta et al (2015) (33)**	Observational: retrospective cohort	USA	2000 - 2006	X	W/B/H/A/I	42221	92.8% (0.6)	16219	824	16956	1040	6489	119	1880	29	677	46
**Hariharan et al (1992) (22)**	Observational: retrospective cohort	USA	1974 - 1990		W/B	18	-	10	1	8	2	-	-	-	-	-	-
**Jorge et al (2019) (34)**	Observational: retrospective cohort	USA	1995 - 2014	X	W/B/A	9080	-	3864	999	4636	1416	-	-	580	100	-	-
**Korbet et al (2007) (23)**	Observational: prospective cohort	USA	1981 - 1998		W/B	75	83.0% (2.8)	54	11	21	9	-	-	-	-	-	-
**Lim et al (2019) (36)**	Observational: prospective cohort	USA	2002 - 2016	X	W/B	1335	89.5% (1.1)	311	76	1024	324	-	-	-	-	-	-
**Raiker et al (2021) (41)**	Observational: retrospective cohort	USA	2020 - 2021	X	W/B	1795	-	1077	26	718	18	-	-	-	-	-	-
**Reveille et al (1990) (20)**	Observational: retrospective cohort	USA	1975 - 1984		W/B	387	-	184	37	203	53	-	-	-	-	-	-
**Serdula et al (1979) (47)**	Observational: retrospective cohort	USA	1970 - 1976		W/A/I	96	88.3% (3.1)	9	2	-	-	-	-	67	22	20	4
**Shah et al (2009) (29)**	Observational: retrospective cohort	USA	1993 - 2002	X	W/B/H	1732	-	1293	105	351	36	88	9	-	-	-	-
**Siegel et al (1969) (19)**	Observational: prospective cohort	USA	1955 - 1968		W/B/H	292	100% (0)	158	102	93	58	41	27	-	-	-	-
**Tan et al (2012) (28)**	Observational: prospective cohort	USA	1979 - 2011		W/B	1979	92.3% (1.4)	1194	54	785	77	-	-	-	-	-	-
**Taylor et al (2023) (38)**	Observational: prospective cohort	USA	2007 - 2017	X	W/B/A	771	-	312	45	164	41	-	-	295	45	-	-
**Ward et al (1995) (21)**	Observational: prospective cohort	USA	1969 - 1991		W/B	361	83.3% (9.2)	184	61	177	78	-	-	-	-	-	-

Study	Study design	Study country	Study period	Registry or database	Racial and Ethnic groups (W, White; B, Black; H, Hispanic; A, Asian; I, Indigenous)	Total number (n)	Female (SD)	White race and ethnicity (n)	White race and ethnicity deaths (n)	Black race and ethnicity (n)	Black race and ethnicity deaths (n)	Hispanic race and ethnicity (n)	Hispanic race and ethnicity deaths (n)	Asian race and ethnicity (n)	Asian race and ethnicity deaths (n)	Indigenous race and ethnicity (n)	Indigenous race and ethnicity deaths (n)
**Ward et al (2002) (25)**	Observational: retrospective cohort	USA	1991 - 1994	X	W/B/H/A/I	9864	-	5690	295	1767	65	1719	87	661	50	27	2
**Wilson et al (2018) (35)**	Observational: retrospective cohort	USA	1998 - 2014		W/B/H/A	238	84.0% (7.3)	78	7	94	17	54	4	12	1	-	-
**Kellum et al (1964) (18)**	Observational: prospective cohort	USA	1949 - 1960		W/B	299	88.4% (4.1)	276	78	23	7	-	-	-	-	-	-
**Fernandez et al (2007) (30)**	Observational: prospective cohort	USA and Puerto Rico	1994 - 2006	X	W/B/H	617	89.3% (4.5)	176	13	221	32	220	18	-	-	-	-
**Ugarte-Gil et al (2023) (40)**	Observational: retrospective cohort	USA and Puerto Rico	2020 - 2021	X	W/B/H	441	91.0% (1.3)	161	5	126	6	154	6	-	-	-	-

Supplementary table 3. Results of meta-regression models; expressed with 95% confidence intervals (CI).

**Study level variables**	**Coefficient [95% CI]**	**P value**
Duration	1.00 [0.99-1.02]	0.95
Mid-year of study period	1.00 [0.99-1.01]	0.68

Supplementary table 4. Risk of bias assessment (Newcastle Ottawa Scale)

Study	Selection	Comparability	Outcome	Summary Score	Risk of bias
Anderson et al. 2008	3	1	2	6	Medium
Azizoddin et al. 2019	3	0	2	5	High
Bruera et al. 2023	3	1	2	6	Medium
Brunner et al. 2006	3	0	2	5	High
Budhoo et al. 2017	2	1	2	5	Medium
Campbell et al. 2008	3	1	3	7	Medium
Carter et al. 2022	3	0	1	4	High
Contreras et al. 2006	3	0	2	5	High
De Castro et al. 2007	3	0	2	5	High
Dooley et al. 1997	3	0	3	6	High
Fernandez et al. 2007	3	1	2	6	Medium
Gomez-Puerta et al. 2015	3	1	2	6	Medium
Hanly et al. 2016	3	1	2	6	Medium
Hariharan et al. 1992	3	0	3	6	High
Hitchon et al. 2007	3	1	2	6	Medium
Johnson et al. 2006	3	1	2	6	Medium
Jorge et al. 2019	3	1	2	6	Medium
Kellum et al. 1964	3	0	2	5	High
Korbet et al. 2007	3	1	3	7	Medium
Lim et al. 2019	3	1	2	6	Medium
Luo et al. 2022	3	0	3	6	High
Nossent et al. 2018	3	0	2	5	High
Raiker et al. 2021	3	0	2	5	High
Reveille et al. 199	3	1	2	6	Medium
Samanta et al. 1991	3	0	2	5	High
Segasothy et al. 2001	3	0	2	5	High
Serdula et al. 1979	3	1	2	6	Medium
Shah et al. 2009	3	1	3	7	Medium
Siegel et al. 1969	3	0	2	5	High
Tan et al. 2012	3	1	2	6	Medium
Taylor et al. 2023	3	0	2	5	High
Ugarte-Gil et al. 2023	3	1	3	7	Medium
Ward et al. 1995	3	1	2	6	Medium
Ward et al. 2002	3	1	2	6	Medium
Wilson et al. 2018	3	1	3	7	Medium
Yee et al. 2015	3	1	3	7	Medium

Supplementary table 5. Risk of bias assessment (Cochrane RoB2 scale)

**Study**	**Randomisation process**	**Allocation concealment**	**Blinding patients and personnel**	**Blinding outcome assessment**	**Incomplete outcome data**	**Selective reporting**
Wallace et al. 2017	Low	Low	Low	Low	Low	Low

Supplementary references

64. Wallace DJ, Strand V, Merrill JT, et al. Efficacy and safety of an interleukin 6 monoclonal antibody for the treatment of systemic lupus erythematosus: a phase II dose-ranging randomised controlled trial. Ann Rheum Dis 2017;76:534–42. 10.1136/annrheumdis-2016-209668

65. Samanta A, Feehally J, Roy S, et al. High prevalence of systemic disease and mortality in Asian subjects with systemic lupus erythematosus. Ann Rheum Dis 1991;50:490–2. 10.1136/ard.50.7.490
